# Artificial intelligence-enhanced volumetric laser endomicroscopy improves dysplasia detection in Barrett’s esophagus in a randomized cross-over study

**DOI:** 10.1038/s41598-022-20610-z

**Published:** 2022-09-29

**Authors:** Allon Kahn, Matthew J. McKinley, Molly Stewart, Kenneth K. Wang, Prasad G. Iyer, Cadman L. Leggett, Arvind J. Trindade

**Affiliations:** 1 Division of Gastroenterology and Hepatology, Mayo Clinic Arizona, Scottsdale, AZ USA; 2 Division of Gastroenterology, Zucker School of Medicine at Hofstra/Northwell, Long Island Jewish Medical Center, 270-05 76th Avenue, New Hyde Park, NY 11040 USA; 3 Institute of Health Innovations and Outcomes Research, Feinstein Institutes for Medical Research, Northwell Health, Manhasset, NY USA; 4 Division of Gastroenterology and Hepatology, Mayo Clinic, Rochester, MN USA

**Keywords:** Oesophageal cancer, Barrett oesophagus

## Abstract

Volumetric laser endomicroscopy (VLE) is an advanced endoscopic imaging tool that can improve dysplasia detection in Barrett’s esophagus (BE). However, VLE scans generate 1200 cross-sectional images that can make interpretation difficult. The impact of a new VLE artificial intelligence algorithm called Intelligent Real-time Image Segmentation (IRIS) is not well-characterized. This is a randomized prospective cross-over study of BE patients undergoing endoscopy who were randomized to IRIS-enhanced or unenhanced VLE first followed by the other (IRIS-VLE vs. VLE-IRIS, respectively) at expert BE centers. The primary outcome was image interpretation time, which served as a surrogate measure for ease of interpretation. The secondary outcome was diagnostic yield of dysplasia for each imaging modality. 133 patients were enrolled. 67 patients were randomized to VLE-IRIS and 66 to IRIS-VLE. Total interpretation time did not differ significantly between groups (7.8 min VLE-IRIS vs. 7 min IRIS-VLE, *P* = 0.1), however unenhanced VLE interpretation time was significantly shorter in the IRIS-VLE group (2.4 min vs. 3.8 min, *P* < 0.01). When IRIS was used first, 100% of dysplastic areas were identified, compared with 76.9% when VLE was the first interpretation modality (*P* = 0.06). IRIS-enhanced VLE reduced the time of subsequent unenhanced VLE interpretation, suggesting heightened efficiency and improved dysplasia detection. It was also able to identify all endoscopically non-visible dysplastic areas.

## Introduction

Barrett’s esophagus (BE) is the precursor to esophageal adenocarcinoma (EAC), a highly fatal malignancy whose incidence continues to rise. EAC develops through increasing grades of dysplasia, which serves as the best-known predictor of cancer progression. Thus, detection and localization of dysplasia in BE is critical to risk stratification and clinical decision making. The current standard practice consists of high-definition white light endoscopy and virtual chromoendoscopy to detect visible lesions, followed by systematic 4-quadrant biopsies throughout the remainder of the BE segment^1^. This approach is limited by sampling error and inability to detect abnormalities below the surface epithelium^2,3^. Recent studies indicate that the current standard practice may result in a 25% rate of missed EAC during index EGD for BE^4^.

Volumetric laser endomicroscopy (VLE) is an endoscopic probe-based application of frequency domain optical coherence tomography that allows for real-time circumferential imaging of the esophageal wall and subsurface structures with a resolution of 7 µm and depth of 3 mm^5^. Several studies have demonstrated the ability of VLE to enhance dysplasia detection and localize abnormalities that were not detected by conventional endoscopic examination^6,7^. However, more widespread implementation of VLE has been limited by the need for the endoscopist to interpret the vast data generated during scans (1200 cross-sectional images per 6 cm scan segment). While various scoring systems have been devised, each requires the endoscopist to correctly identify imaging features. In response, an artificial intelligence algorithm called Intelligent Real-time Image Segmentation (IRIS) has been developed and incorporated into the VLE console system to automatically highlight VLE features known to be associated with dysplasia^8^ . These include epithelial glands, loss of layering, and increased surface signal intensity. A similar, recently-developed computer-aided detection (CAD) algorithm was developed and tested on targeted regions of interest (ROI), showing an impressive 85% accuracy in neoplasia detection^9^.

The purpose of the IRIS system is to ease the burden of interpretation on the endoscopist and facilitate dysplasia detection. However, the performance of IRIS has not been comparatively assessed against un-enhanced VLE scans, both in terms of dysplasia detection and time of image interpretation. The principle aim of this randomized cross-over study was to assess the incremental effect of IRIS enhanced VLE with regard to time of interpretation and diagnostic yield. We hypothesized that IRIS would enhance dysplasia detection and reduce interpretation time by highlighting ROI’s within the VLE scan.

## Methods

### Patient selection

Patients with BE segments ≥ 2 cm in length undergoing upper endoscopy and clinically-indicated VLE for surveillance were approached for enrollment in the trial between January 23rd 2019 and March 26th 2020 at three tertiary referral academic medical centers with extensive experience in VLE and BE endoscopy. Patients with severe erosive esophagitis or esophageal stenosis precluding safe use of VLE were excluded. Patients were also excluded if they were under the age of 18 years old or had any contraindications to undergo a clinically indicated endoscopy. All patients provided written informed consent prior to study participation. The study was approved by the Northwell Health Institutional Review Board (IRB), along with the IRB at each participating institution and all methods were performed in accordance with the relevant guidelines and regulations. The study was registered on January 24th, 2019 with the National Institutes of Health under clinical trial #NCT03814824.

### Endoscopic assessment, randomization and allocation of study groups

All study-related endoscopic assessments, VLE scans, and VLE interpretation were carried out by expert users, defined as having performed and interpreted at least 100 VLE scans. Patients enrolled in the study underwent clinically indicated endoscopy for BE surveillance. An initial visual examination was performed to determine the presence and absence of endoscopic inclusion and exclusion criteria respectively. Once eligibility was verified, patients were randomized 1:1 using an electronic randomization system to undergo IRIS-enhanced or unenhanced VLE followed by the counterpart imaging modality. As a result, the following groups were defined:

1. **VLE-IRIS:** Patients allocated to the VLE-IRIS group underwent imaging by VLE with the IRIS software disabled followed by a second interpretation with the IRIS imaging enabled.
2. **IRIS-VLE:** Patients allocated to the IRIS-VLE group underwent imaging and interpretation with the IRIS software enabled followed by a second interpretation by VLE with IRIS disabled.

**Figure 1:**
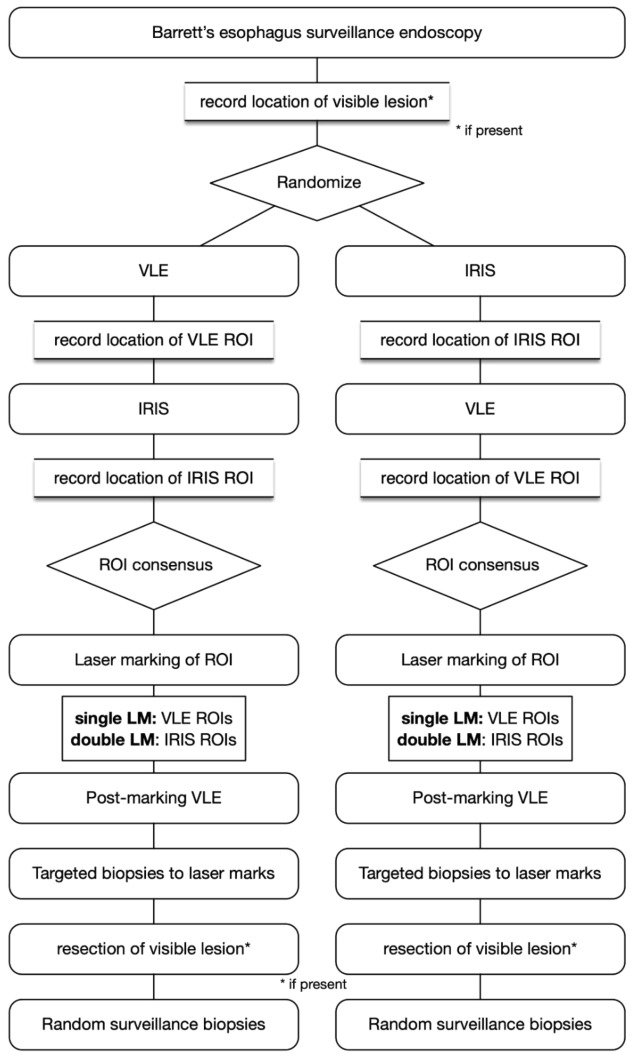
Flow chart of patient allocation and study procedures. ROI—Region of interest; VLE—volumetric laser endomicroscopy; IRIS—Intelligent Real-Time Image Segmentation.

A flow chart depicting the study process is demonstrated in Fig. 1. Prior to performing VLE imaging, a detailed endoscopic examination was then carried out using both high-definition white light (HDWLE) and narrow-band imaging (NBI). The presence and size of a hiatal hernia was recorded. The length of the BE segment was measured and reported in standard terminology using the Prague classification. In cases where visible lesions were present, the location of the lesion was documented in centimeters from the incisors and the orientation of the lesion was recorded using clock face positioning with the gastroscope in the neutral position. The morphology of the lesion was classified using the Paris classification.

### VLE scan protocol

The VLE platform (NVisionVLE® system, NinePoint Medical Inc., Bedford, MA) consists of a console, monitor, and balloon probe. Balloon probes are 6 cm in length and are available in 14 mm, 17 mm, and 20 mm diameters. The selection of the appropriate balloon diameter was made by the performing endoscopist according to the endoscopic inspection and estimation of esophageal diameter. The balloon probe was connected to the console and then passed through the working channel of the gastroscope. The balloon was then centered in the esophageal lumen with the distal tip located at least 1 cm distal to the gastroesophageal junction (GEJ). The balloon was inflated to 15 psi to anchor its position and center the optical probe. Manual scanning was then initiated to allow for real-time dynamic imaging. The balloon position was adjusted to optimize centering of the optical probe. The optical probe was then retracted into the static balloon until the position of the balloon relative to the GEJ could be verified. A full VLE scan was then obtained, consisting of automated helical pullback of the optical probe through the entire 6 cm balloon over 90 s. Additional overlapping scans were performed as needed to encompass the entire BE segment.

A full 6 cm VLE scan consists of 1200 frames that can be displayed in circumferential, longitudinal, and en-face orientation with the ability to magnify areas of interest in real time. The VLE console enables the user to review the scan in realtime. The IRIS system is a software enhancement that is toggled via dedicated buttons on the VLE user interface. The system uses a machine learning algorithm to automatically detect the three VLE imaging features known to be associated with dysplasia and highlights them via a color-graded overlay: (1) increased surface signal intensity (pink), (2) abnormal epithelial glands (blue), and (3) loss of mucosal layering (orange).

The interpreting endoscopist was asked to identify any areas of concern based on subjective assessment of established imaging features associated with dysplasia^10–12^. The location of these areas was recorded according to numerical clock face and longitudinal frame position as noted on the VLE console. Any two regions of interest (ROI) within 75 longitudinal frames or 2 h’ clock face position were considered to be the same target. Identification of only one ROI per centimeter was allowed in order to avoid overlap. The time of interpretation was recorded for each imaging modality.

Once VLE interpretation was completed, all ROI’s underwent marking using the VLE laser marking system. VLE laser marking involves the use of a low-power laser beam channeled through the optical probe and engaged through the use of a handheld trigger. VLE laser marking generates superficial cautery marks in the esophageal mucosa and can be configured as single or double marks. ROI’s that were identified by only unenhanced VLE were marked with a single laser mark. Those identified only by IRIS were denoted with a double laser mark. If the modalities agreed (i.e. the ROI was identified by both unenhanced VLE and IRIS-enhanced VLE), a double laser mark was used and this was recorded. After laser marking, a final scout scan (600 frames) was obtained to verify accurate laser marking position of the ROI. The balloon probe was then deflated and removed. Targeted biopsies were obtained at each laser marked area with a large-capacity biopsy forceps and placed in separate specimen containers. Resection of any previously identified visible lesions was then performed. Finally, random biopsies were taken from the remainder of the BE segment at set interval as per the standard surveillance protocol. All tissue specimens sent for histology were reviewed by experienced gastrointestinal pathologists who graded highest degree of dysplasia.

### Outcome measures

The primary outcome in this study was time of image interpretation. This served as a surrogate measure of ease of interpretation. The time was recorded in total and was also defined by the length of the BE segment as time per cm of BE. The secondary outcomes included diagnostic yield of dysplasia for each imaging modality and the number of biopsies required to make a diagnosis of dysplasia. For the latter metric, it was assumed that two biopsies were taken at each laser-marked ROI, as is our standard practice. The number of biopsies was utilized as a secondary outcome to measure diagnostic efficiency, or the number of ROI’s needed to make a diagnosis of dysplasia.

### Power statement

The expected mean time for a VLE without IRIS is 5 min + /− 3 min. With a CI of 95% a sample size for 1:1 randomization of 94 (47 in each group) would have 90% power to detect a difference of 2 min.

### Statistical methods

Categorical and numerical variables are summarized as counts (percents) and mean (standard deviation), respectively. The chi-square test was used to compare the distribution of categorical variables between study groups, while the Wilcoxon Rank Sum test was used to compare the distribution of numerical variables. To account for each patient being evaluated with each technology, a general linear model (GLM) with a binomial distribution and logit link was implemented with a random intercept to estimate the relative change in odds of detecting dysplasia for one technology, such as IRIS or WLE/NBI, relative to another. All hypotheses tested were two-sided with *p* < 0.05 considered statistically significant. Analyses were performed in SAS v9.4 (SAS Institute, Inc.; Cary, NC).

## Results

### Study population

A total of 133 patients were enrolled and completed the study-related procedures. The demographic details and clinical features of the study population are detailed in Table 1. 67 patients were randomized to the VLE-IRIS group and 66 to the IRIS-VLE group. Although the pre-specified accrual target for the primary endpoint was met, additional patients were enrolled to increase the number of patients with dysplasia. Clinical variables were balanced between study cohorts, except for the presence of a hiatal hernia, which was significantly more likely in the VLE-IRIS group (83.6% vs. 68.2%, *p* = 0.04). The majority of patients (75.2%) had a prior history of dysplasia, with no significant difference between groups. There were four device malfunctions, but none resulted in an adverse event.

**Table 1:** Patient demographics and clinical characteristics.

	Study arm		*P* value
	VLE-IRIS (N = 67)	IRIS-VLE (N = 66)	
**Age,** mean (SD)	64.1 (12.60)	66.1 (11.39)	0.41
**Male sex**, n (%)	43 (64.2%)	48 (72.7%)	0.29
**Years since BE diagnosis,** mean (SD)	4.9 (6.18)	3.1 (3.57)	0.16
**Highest prior grade of dysplasia**, n (%)			0.41
NDBE	20 (29.9%)	11 (17.2%)	
IND	13 (19.4%)	12 (18.8%)	
LGD	17 (25.4%)	24 (37.5%)	
HGD	13 (19.4%)	14 (21.9%)	
EAC	4 (6.0%)	3 (4.7%)	
Unknown	0	2 (3%)	
**Hiatal Hernia**, n (%)	56 (83.6%)	45 (68.2%)	**0.04**
**Hiatal Hernia Length (cm)**, mean (SD)	2.6 (1.29)	2.9 (1.94)	0.82
**Device malfunction**, n (%)	2 (3.0%)	2 (3.0%)	0.99
**Length of BE segment (C)**, mean (SD)	1.7 (2.56)	2.6 (3.48)	0.24
**Length of BE segment (M)**, mean (SD)	3.9 (2.63)	4.5 (3.21)	0.32
**Dysplasia present**, n (%)	17 (25.4%)	19 (28.8%)	0.8
**Total # VLE scans**^**b**^, mean (SD)	3.0 (1.38)	2.9 (1.50)	0.51
**Total procedure time**^**a**^ (min), mean (SD)	54.6 (18.65)	53.0 (20.88)	0.43

Significant values are in bold.

*SD* standard deviation, *NDBE* non-dysplastic Barrett’s esophagus, *IND* Barrett’s esophagus indefinite for dysplasia, *LGD* low-grade dysplasia, *HGD* high-grade dysplasia, *EAC* Esophageal adenocarcinoma, *C* circumferential, *M* maximal, *VLE* volumetric laser endomicroscopy, *IRIS* intelligent real-time image segmentation.

^a^Procedure time was defined as time from scope insertion to removal and includes endoscopic therapy and all endoscopic sampling in addition to imaging.

^b^During the single endoscopy session.

### Primary outcome

The unenhanced VLE and IRIS-enhanced VLE interpretation time metrics for the primary study outcome are shown in Table 2. While the total interpretation time did not differ significantly between groups, the unenhanced VLE interpretation time was significantly shorter in the IRIS-VLE group. In further subdividing first and second interpretation between study groups, second interpretation was significantly shorter in the VLE-IRIS group, even when adjusted for the length of the BE segment. This contrasts with the first interpretation time, which did not differ between groups, even when scaled for BE length. This suggests that less time is needed for evaluation of unenhanced VLE after first reviewing images enhanced by the IRIS software.

**Table 2:** VLE interpretation times between study groups.

	Study arm		*P*-value
	VLE-IRIS (N = 67)	IRIS-VLE (N = 66)	
**Total interpretation time (VLE/IRIS) (min)**			0.1
Mean (SD)	7.8 (5.1)	7.0 (5.2)	
Median	6.0	5.0	
**Interpretation time (VLE alone) (min)**			** < .01**
Mean (SD)	3.8 (2.08)	2.4 (2.10)	
Median	3.0	1.5	
**First interpretation Time (min)**			0.43
Mean (SD)	3.8 (2.08)	4.6 (3.43)	
Median	3.0	4.0	
**Scaled first interpretation time (cm/min)**			0.96
Mean (SD)	1.2 (0.86)	1.1 (0.62)	
Median	1.0	1.0	
**Second interpretation time (min)**			** < .01**
Mean (SD)	4.0 (3.75)	2.4 (2.10)	
Median	3.0	1.5	
**Scaled second interpretation time (cm/min)**			** < .01**
Mean (SD)	1.3 (1.25)	0.6 (0.47)	
Median	1.0	0.5	

All time values are displayed as minutes, except as otherwise noted. All P-values are based on the Kruskal–Wallis test. Scaled interpretation time denotes time of interpretation as a function of BE length in cm.

Significant values are in bold.

*SD* standard deviation, *VLE* volumetric laser endomicroscopy, *IRIS* intelligent real-time image segmentation.

#### Secondary outcomes

**Figure 2:**
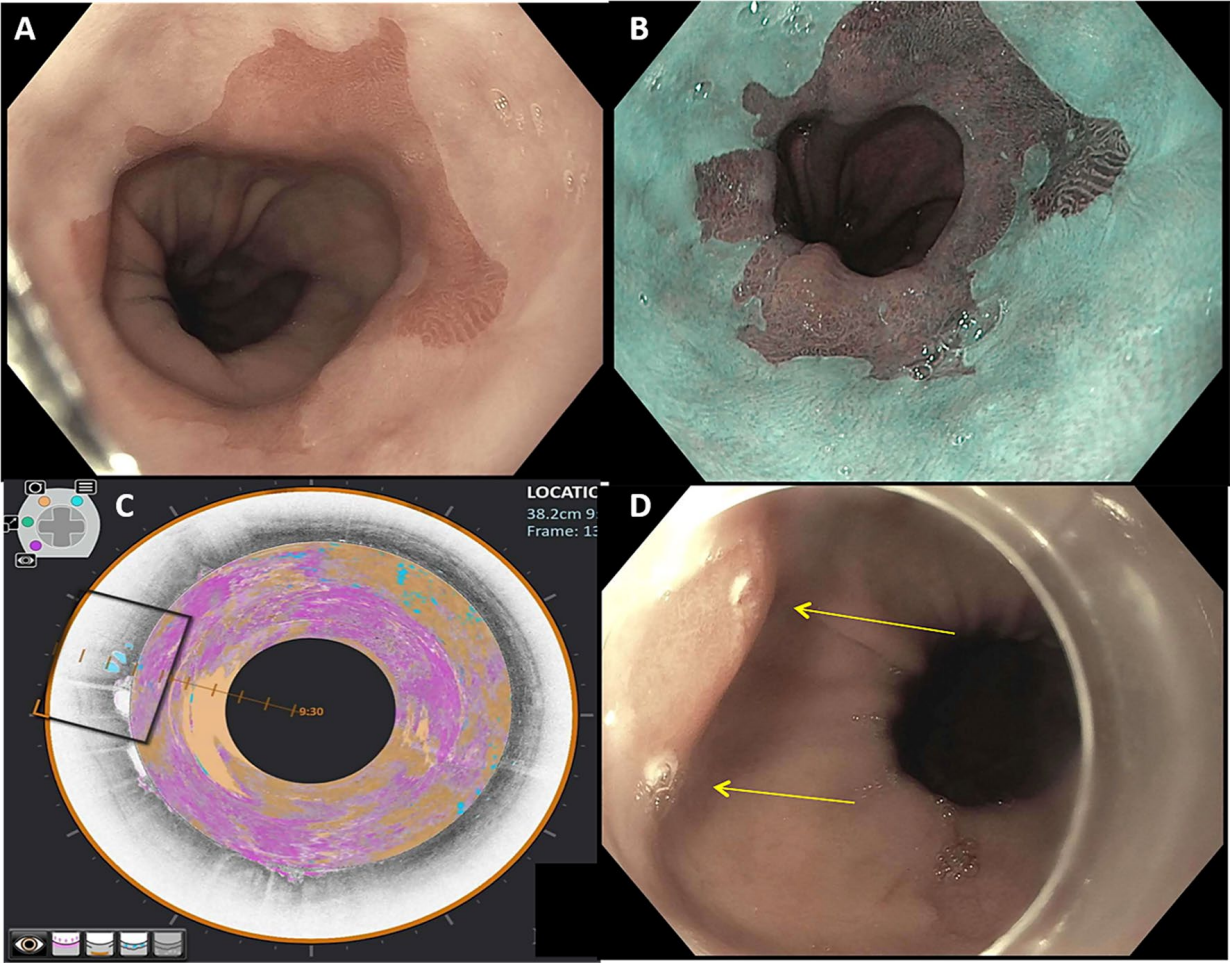
Example of a VLE-IRIS case with endoscopically non-visible dysplasia. Case images from a patient with no endoscopic evidence of dysplasia on (**A**) high-definition white light endoscopy and (**B**) narrow-band imaging. (**C**) VLE with IRIS demonstrates atypical epithelial glands (blue), loss of layering (orange), and increase surface signal intensity (pink). (**D**) Laser marks (yellow arrows) placed during VLE at the site of the abnormality. Biopsies between the laser marks showed high-grade dysplasia.

Dysplasia or early neoplasia was detected in 36 patients during the study procedure. A case example with unenhanced VLE and IRIS enhancement is shown in Fig. 2. 12 of these patients (5 in VLE-IRIS and 7 in IRIS-VLE groups) had dysplasia that was visible under HDWLE and/or NBI and therefore also underwent endoscopic resection. There was no difference in the overall diagnostic yield for dysplasia between unenhanced VLE and IRIS-enhanced VLE (OR 1.39, 95% CI 0.36–5.4, *P* = 0.92) when each modality was evaluated independently, irrespective of interpretation order. There was also no significant difference in diagnostic yield for dysplasia when comparing IRIS-enhanced (OR 3.15, 95% CI 0.8–12.32, *P* = 0.13) or unenhanced VLE (OR 2.26, 95% CI 0.61–8.4, *P* = 0.37) to the endoscopic examination (HDWLE/NBI). When IRIS-enhanced VLE was used as the first interpretation modality, 100% of dysplastic ROI’s were identified, compared with 76.9% when unenhanced VLE was used as the first interpretation modality (*P* = 0.06). The number of biopsies required to make a diagnosis of dysplasia also did not differ significantly between unenhanced VLE and IRIS-enhanced VLE (4.9 ± 2.6 vs. 5.4 ± 2.5 biopsies, *P* = 0.55), even after adjustment for BE length (1.1 ± 0.8 vs. 1.3 ± 0.8 biopsies, *P* = 0.21).

## Discussion

The accurate detection and localization of dysplasia in BE is critical to determine risk and the potential of endoscopic eradication. While VLE enhances the endoscopist’s ability to achieve these important tasks, the resulting image data sets are large, complex, and require expertise to interpret. In this randomized study, the addition of the artificial intelligence IRIS software platform was able to identify all endoscopically non-visible dysplastic ROI’s and reduce the time of subsequent unenhanced VLE interpretation, suggesting heightened efficiency and improved dysplasia detection. These findings are a powerful demonstration of how machine learning tools can augment the user experience of increasingly complex imaging tools so that they can be leveraged to improve patient outcomes.

The use of unenhanced VLE has previously been demonstrated to significantly increase the diagnostic yield of targeted biopsies for dysplasia when compared to Seattle protocol biopsies. In a study of 448 consecutive BE patients, the dysplasia yield was 33.7% in VLE with laser marking, compared with only 19.6% in the Seattle protocol biopsy group (odds ratio 2.1, *P* = 0.03)^6^. However, unenhanced VLE requires the endoscopist to have both the expertise and time to review and interpret a large amount of data. These factors have impeded widespread adoption.

Initial studies establishing the use of unenhanced VLE in BE patients indicated a mean VLE procedure time of 22 min^13^. However, this accounted for only aspects of the actual procedure, including balloon positioning, inflation, deflation, and other non-interpretation tasks. Only one prior study has reported assessment time of full unenhanced VLE scan data using a web-based platform to examine diagnostic accuracy in expert users^14^. The median interpretation time by VLE experts was 6.5 min. However, the web-based platform did not match the user interface of the VLE console device, so differences in the scan interpretation timing would be expected. The present study represents a much more robust assessment of interpretation time in real-world clinical practice, showing that the mean interpretation time was approximately 4 min.

Even in expert hands, studies of VLE interpretation have shown a wide range of inter-rater reliability, with Kappa values ranging from 0.28 to 0.82^15,16^. This and issues about time constraints led to the advent of the IRIS platform to enhance the ease of interpretation. In this study, our primary outcome was interpretation time as a surrogate measure for ease of interpretation. By randomizing the order of interpretation, it was possible to determine the time of interpretation for each imaging modality (IRIS-enhanced or unenhanced VLE) without internal bias. The total interpretation time did not differ between groups. However, when patients were randomized to undergo IRIS first, the subsequent time of interpretation for unenhanced VLE was significantly reduced. There are several plausible reasons for this effect. The first task faced by the endoscopist in VLE interpretation is localization of any abnormality. IRIS provides a color-graded “en face” reconstruction of the entire imaged esophageal segment that facilitates rapid identification of abnormal signal patterns. It is possible that seeing this overview enhanced endoscopist confidence that no other abnormal areas would be identified on the unenhanced VLE review. The second task is to examine these areas and determine whether they meet criteria for suspected dysplasia. IRIS also assists in this task by providing color-graded overlays of abnormal imaging features. Again, it is possible that the interpreting endoscopist experienced enhanced confidence in diagnosis and did not feel the need to spend as much time reviewing the unenhanced VLE images.

There are limited data on the role of IRIS-enhanced VLE in dysplasia detection. In a study of 71 laser-marked ROI’s with histologic correlation, various diagnostic scoring systems were used by expert raters with and without IRIS enhancement to diagnose dysplasia [16]. The diagnostic accuracy was not increased by the use of IRIS in expert users. However, this analysis did not assess ROI identification or localization. In the present study, IRIS did not result in overall increased dysplasia yield in expert users. However, when visible lesions were excluded and only dysplastic ROI’s were analyzed, IRIS-enhanced VLE was able to detect 100% of dysplastic ROI’s compared to 76.9% for unenhanced VLE. This comports with a prior study of unenhanced full VLE scans assessed by expert users, in which 73% of dysplastic scans were correctly labeled as such. While this difference did not reach statistical significance in our study, it suggests that adding IRIS to these scans may have further enhanced localization of the known dysplastic ROI’s and therefore that IRIS may generally enhance dysplasia localization.

While IRIS is one proposed solution to the challenge of VLE data interpretation, others have also been developed. The PREDICT study utilized convolutional neural networks to develop and validate a computer aided detection (CAD) algorithm for BE dysplasia detection^9^. In the independent testing set, the derived algorithm demonstrated an accuracy of 85%, sensitivity of 91% and specificity of 82% for the diagnosis of dysplasia. The PREDICT study utilized laser-marked VLE targets for both the development and validation of the algorithm. In contrast, our study utilized real-time full scan interpretation, which more closely approximates clinical practice. Clinical care in BE is typically driven by the highest level of pathology. Despite the fact that the PREDICT algorithm exhibited an 85% accuracy in the test set, it identified all patients with neoplasia. Therefore both the PREDICT and IRIS algorithms accomplished the most important clinical goal of a BE examination. However, unlike IRIS, the PREDICT algorithm has not been validated in real-time full scan interpretation.

Our study has several strengths. It was conducted prospectively with randomization of patients to reduce internal bias between image interpretation modalities. The study endoscopists are established experts in VLE interpretation and BE endoscopy. The pre-specified accrual targets were met and exceeded, indicating adequate power for statistical comparisons.

We also acknowledge several important limitations. The study population consisted of patients referred to expert centers and this may reduce external generalizability. By utilizing expert users, we were not able to assess the impact of IRIS on novice interpretation. We also utilized a surveillance biopsy protocol that avoided areas of laser marking to avoid confounding and were thus unable to meaningfully compare VLE-related dysplasia detection to that which would have occurred in the standardized biopsy protocol. Finally, although we did randomize the order of interpretation, it is not possible to eliminate all bias from a subjective interpretation paradigm.

In conclusion, we conducted a randomized cross-over study of IRIS-enhanced and unenhanced VLE to assess the impact of IRIS on the ease and accuracy of VLE interpretation. We demonstrated that IRIS reduced the time needed for second interpretation, likely by increasing endoscopist confidence in lesion localization and diagnosis. Additionally, IRIS enhancement was able to detect all non-visible dysplastic ROI’s, suggesting it may play an important role in ROI identification. Further studies are needed to confirm the reproducibility of the observation that IRIS enhancement of VLE scans may improve efficiency and confidence of the interpreting endoscopist.

## Data Availability

The data that support the findings of this study are available from the corresponding author, AJT, upon request.
